# Six Novel Mycoviruses Containing Positive Single-Stranded RNA and Double-Stranded RNA Genomes Co-Infect a Single Strain of the *Rhizoctonia solani* AG-3 PT

**DOI:** 10.3390/v14040813

**Published:** 2022-04-14

**Authors:** Yuting Li, Siwei Li, Yumeng Zhao, Tao Zhou, Xuehong Wu, Can Zhao

**Affiliations:** 1 College of Plant Protection, China Agricultural University, Beijing 100193, China; liyuting990508@163.com (Y.L.); lisiwei28@163.com (S.L.); 18811774047@163.com (Y.Z.); taozhoucau@cau.edu.cn (T.Z.); wuxuehong@cau.edu.cn (X.W.); 2 College of Horticulture, China Agricultural University, Beijing 100193, China

**Keywords:** co-infection, fusarivirus, *Alphapartitivirus*, Bipartitiviridae, RNA-dependent RNA polymerase, rapid amplification of cDNA ends, *Rhizoctonia solani*, metatranscriptome

## Abstract

Six novel mycoviruses that collectively represent the mycovirome of *Rhizoctonia solani* anastomosis group (AG)-3 PT strain ZJ-2H, which causes potato black scurf, were identified through metatranscriptome sequencing and putatively designated as Rhizoctonia solani fusarivirus 4 [RsFV4, positive single-stranded RNA (+ssRNA)], Rhizoctonia solani fusarivirus 5 (RsFV5, +ssRNA), Rhizoctonia solani mitovirus 40 (RsMV40, +ssRNA), Rhizoctonia solani partitivirus 10 [RsPV10, double-stranded RNA (dsRNA)], Rhizoctonia solani partitivirus 11 (RsPV11, dsRNA), and Rhizoctonia solani RNA virus 11 (RsRV11, dsRNA). Whole genome sequences of RsFV4, RsMV40, RsPV10, RsPV11, and RsRV11, as well as a partial genome sequence of RsFV5, were obtained. The 3’- and 5’- untranslated regions of the five mycoviruses with complete genome sequences were folded into stable stem-loop or panhandle secondary structures. RsFV4 and RsFV5 are most closely related to Rhizoctonia solani fusarivirus 1 (RsFV1), however, the first open reading frame (ORF) of RsFV4 and RsFV5 encode a hypothetical protein that differs from the first ORF of RsFV1, which encodes a helicase. We confirmed that RsPV10 and RsPV11 assemble into the spherical virus particles (approximately 30 nm in diameter) that were extracted from strain ZJ-2H. This is the first report that +ssRNA and dsRNA viruses co-infect a single strain of *R. solani* AG-3 PT.

## 1. Introduction

Mycoviruses have been identified in a wide range of fungal species [1,2]. Generally, mycovirus infection is symptomless, without any discernable phenotypic alterations visible in the infected host [3,4]. This feature sometimes prevents the discovery of mycoviruses and has limited progress in mycoviral research. Metatranscriptome sequencing is sensitive to low titer viral infections and has been widely used to discover viruses in different environments or hosts [5,6,7,8]. Metatranscriptome sequencing can also alter the diagnosis of a singular infection in fungi to a cryptic co-infection [6,9]. In fact, metatranscriptome sequencing has revealed diverse mycoviruses co-infecting a single isolate of a fungal host and demonstrated that this is a common occurrence [2,10,11,12,13,14].

*Rhizoctonia solani* Kühn [teleomorph: *Thanatephorus cucumeris* (Frank) Donk] is a soil-borne, cosmopolitan basidiomycetous fungus that can infect a wide range of crops, including rice, maize, wheat, and potato, causing significant economic losses [15,16,17,18]. Stem canker or black scurf of potato is caused by multinucleate and binucleate *Rhizoctonia*, including *R. solani* anastomosis group (AG)-1, AG-2, AG-3 PT, AG-4HGI, AG-4HGII, AG-4HGIII, AG-5, AG-6, AG-7, AG-8, AG-9, AG-10, and AG-11, and *Ceratobasidium* AG-A, AG-F, AG-G, AG-I, AG-K, and AG-W [18,19,20,21,22,23]. Notably, AG-3 PT is widely considered the predominant and the most aggressive of the AGs in potato plants [24,25,26].

Since the first mycovirus was detected in *R. solani* in 1978 [27], more than 100 mycoviruses have been identified in this pathogenic fungus [28,29,30,31,32]. To date, the mycoviruses identified in *R. solani* are double-stranded RNA (dsRNA), positive single-stranded RNA (+ssRNA), or negative single-stranded RNA (−ssRNA) viruses, and include members of established families as well as members of proposed families and unclassified RNA viruses [11], such as Rhizoctonia solani virus 717 (dsRNA, *Partitiviridae*) [33], Rhizoctonia solani parititivirus 2 (dsRNA, *Partitiviridae*) [34], Rhizoctonia solani fusarivirus 1 (+ssRNA, proposed family Fusariviridae) [35], Rhizoctonia solani mitovirus 39 (+ssRNA, *Mitoviridae*) [36], Rhizoctonia solani negative-stranded virus 1 (−ssRNA, proposed family Betamycoserpentoviridae) [12], Rhizoctonia solani mycovirus 2 (dsRNA, proposed family Bipartitiviridae), etc. [37]. Quite a few of the mycoviruses in *R. solani,* however, are incompletely sequenced [12,31,32,35].

Co-infection by multiple mycoviruses has been reported in *R. solani*, with the co-infecting mycoviruses typically representing members within the same family, such as partitiviruses (family *Partitiviridae*) found in *R. solani* AG-3 TB and AG-1-IA [38,39], or mitoviruses (family *Mitoviridae*) found in *R. solani* AG-3 PT [32]. Strikingly, deep sequencing analysis revealed that at least 17 viruses co-infected an avirulent isolate of *R. solani* AG-2-2IV isolated from sugar beet, and included members of the families *Mitoviridae*, Fusariviridae, *Endornaviridae*, *Partitiviridae*, *Amalgaviridae*, *Megabirnaviridae*, and unclassified viruses [37]. Notably, however, natural co-infection of a single strain of *R. solani* AG-3 PT, the causal agent of stem canker or black scurf of potato, by +ssRNA and dsRNA viruses has not been reported.

In the present study, multiple bands of dsRNA were detected in *R. solani* AG-3 PT strain ZJ-2H, originally isolated from potato tubers with symptoms of black scurf planted in Wenzhou city, Zhejiang province, China [18]. We demonstrated, using metatranscriptome sequencing, that six novel mycoviruses (three +ssRNA viruses and three dsRNA viruses) comprise the mycovirome of *R. solani* AG-3 PT strain ZJ-2H. Whole genome sequences of five mycoviruses and a partial genome sequence of one mycovirus were assembled by combining the sequences obtained by RNA sequencing (RNA-Seq) with the terminal sequences obtained using rapid amplification of cDNA ends (RACE). The genomic and phylogenetic features of the six mycoviruses are presented to characterize the mycovirome of strain ZJ-2H.

## 2. Materials and Methods

### 2.1. Extraction and Purification of RNA

*R. solani* AG-3 PT strain ZJ-2H was cultured on potato dextrose agar (PDA) plates with cellophane film membranes (PDA-CF) at 25 °C in the dark for five days for subsequent extraction of dsRNA or total RNA. Approximately 0.2 g fresh mycelia were collected from PDA-CF plates and ground to a fine powder in liquid nitrogen. Double-stranded RNA of strain ZJ-2H was extracted using the CF-11 cellulose (Sigma-Aldrich, St. Louis, MI, USA) chromatography method as previously described [40,41] with minor modifications. For purification, dsRNA suspensions were treated with DNase I and S1 Nuclease (TaKaRa, Dalian, China) to remove genomic DNA and ssRNA, respectively. The purified dsRNA was then assessed using 1% agarose gel electrophoresis. Total RNA was extracted from strain ZJ-2H using TRIzol Reagent (Invitrogen, Carlsbad, CA, USA) according to the manufacturer’s instructions, and used for complementary DNA (cDNA) synthesis and metatranscriptome sequencing.

### 2.2. Metatranscriptome Sequencing

RNA-sequencing (RNA-Seq) of *R. solani* AG-3 PT strain ZJ-2H was conducted by Shanghai Biotechnology Corporation using an Illumina HiSeq 2500 platform for paired-end sequencing. Sequencing libraries were established from rRNA-depleted total RNA samples of strain ZJ-2H using a TruSeq Stranded Total RNA LT Sample Prep Kit (Illumina, Inc., San Diego, CA, USA). Library quality assessment was carried out on a Qubit^®^ 2.0 Fluorometer (Invitrogen, Q32866) and Agilent Technogies 2100 Bioanalyzer (Agilent Technogies Inc., La Jolla, CA, USA). The resulting raw reads were filtered to obtain high quality clean reads. A de novo assembly of the sequences was constructed using CLC Genomics Workbench version 6.0.4 software. The resulting unigenes were queried against the National Center for the Biotechnology Information (NCBI) non-redundant (NR) database and aligned using BLASTx to obtain homologous viral sequences of mycoviruses.

### 2.3. Validation of Mycoviruses in Strain ZJ-2H

Reverse transcription–polymerase chain reaction (RT-PCR) was performed using specific primer pairs based on the nucleotide sequences of RNA-dependent RNA polymerase (RdRp), coat protein (CP), or hypothetical protein (HP) of the six mycoviruses (Table S1) to confirm the presence of the identified viruses in *R. solani* AG-3 PT strain ZJ-2H. Total RNA of strain ZJ-2H was reversely amplified to synthesize first-strand cDNA using moloney murine leukemia virus (M-MLV) reverse-transcriptase and random primer pd(N)_6_ (TaKaRa, Dalian, China) according to the manufacturer’s instructions. The amplified products were then used as a template in subsequent RT-PCR amplifications. The 25 µL RT-PCR reaction mixture consisted of 18.3 µL ddH_2_O, 2.5 µL 10 × *Ex Taq* buffer (Mg^2+^ plus, 20 mM), 2.0 µL dNTP mixture (2.5 mM for each), 0.5 µL of each primer pair (10 µM), 0.2 µL *Ex Taq* (5 U/µL, TaKaRa), and 1.0 µL cDNA template. RT-PCR was performed in an Eppendorf Mastercycler gradient thermal cycler (Eppendorf, Hamburg, Germany) using the following program: initial denaturation at 94 °C for 3 min; followed by 30 cycles of denaturing at 94 °C for 30 s, annealing at temperature of the primer pairs listed in Table S1 for 30 s, and extension at 72 °C for 60 s; and a final extension at 72 °C for 10 min. After examination of the PCR products using 1% agarose gel electrophoresis, amplicons of the expected length were purified using a gel extraction kit (Axygene Biosciences, Union City, CA, USA) and then cloned into pClone007 Versatile Simple Vector (TsingKe, Beijing, China). The cloning reaction mixtures were transformed into *Escherichia coli* Top 10 cells (Aidlab Biotechnologies, Beijing, China) by heat shock at 42 °C for 90 s, and then cultured on Luria-Bertani broth media containing ampicillin. Colonies verified by PCR were selected and sent to Beijing Tianyihuiyuan Co., Ltd. (Beijing, China) for Sanger sequencing.

### 2.4. Determination of Full Length cDNAs of Putative Mycoviruses

RT-PCR was performed using specific primer pairs that were designed based on the sequences of the generated viral contigs (Table 1) to verify the sequences obtained by RNA-Seq. At least two primer pairs (Table S2) were designed for each putative mycovirus. RT-PCR and Sanger sequencing were performed as described in Section 2.3.

The 5’- and 3’-terminal sequences of the putative mycoviruses were determined by the RACE method using a SMARTer RACE 5’/3’ Kit (TaKaRa) according to the manufacturer’s instructions. The amplified products were ligated into the pClone007 Versatile Simple Vector (TsingKe, Beijing, China) and sequenced by Beijing Tianyihuiyuan Co., Ltd. (Beijing, China). The identity of each base was determined using at least three independent clones that were sequenced in both directions. The primers used to determine the terminal sequences of the mycoviruses in this study are listed in Table S3.

The viral genome sequence obtained by combining the entire sequence resulting from RNA-Seq and the terminal sequence obtained using the RACE method was assembled using DNAMAN version 7.0. The confirmed complete genome sequence of five mycoviruses and partial genome sequence of one mycovirus were submitted to NCBI.

### 2.5. Sequence Analysis

The open reading frames (ORFs) were predicted using the ORF finder program at NCBI based on the standard genetic code or fungal mitochondrial genetic code. BLASTp and BLASTx programs were used to search for homology with viral sequences contained in the NCBI NR database. A search for conserved motifs was conducted using the Conserved Domain Database (CDD) (http://www.ncbi.nlm.nih.gov/Structure/cdd/wrpsb.cgi (accessed on 19 January 2021)), the Protein family (Pfam) database (http://pfam.sanger.ac.uk/ (accessed on 19 January 2021)), and the PROSITE database (http://www.expasy.ch/ (accessed on 19 January 2021)). CLUSTAL_X was used to perform sequence alignments [42]. Phylogenetic trees were constructed with MEGA software version 6.0 using the maximum likelihood (ML) method in a Jones–Taylor–Thornton (JTT) model with 1000 bootstrap replicates [43]. The secondary structures of 5’- and 3’-untranslated regions (UTR) of putative mycoviruses were predicted using the RNAfold Webserver (http://rna.tbi.univie.ac.at/cgi-bin/RNAWebSuite/RNAfold.cgi (accessed on 19 September 2021)).

### 2.6. Extraction and Observation of Virus Particles

*R. solani* AG-3 PT strain ZJ-2H was cultured on PDA-CF plates for five days in the dark at 25 °C. Approximately 30 g of fresh mycelia were collected and ground to a fine powder in liquid nitrogen. The mycelial powder was then used to extract virus particles, as previously described [36]. The obtained virus particles were suspended in 100 μL phosphate buffer saline (PBS, 0.05 M, pH 7.2), stained with 1% (*w*/*v*) uranyl acetate, and observed with a transmission electron microscope (TEM, JEM-1230, JEOL, Tokyo, Japan).

### 2.7. Confirmation of the Mycoviruses That Assemble into Virus Particles

To confirm which virus was responsible for the formation of virus particles extracted from *R. solani* AG-3 PT strain ZJ-2H, total RNA was extracted from the virus particles using TRIzol Reagent (Invitrogen) according to the manufacturer’s instructions and reversely amplified to synthesize first-strand of cDNA according to the methods described in Section 2.3. The resulting PCR product was then used as a template for RT-PCR. RT-PCR was performed as previously described in Section 2.3.

## 3. Results

### 3.1. Identification of Mycoviruses

**Figure 1:**
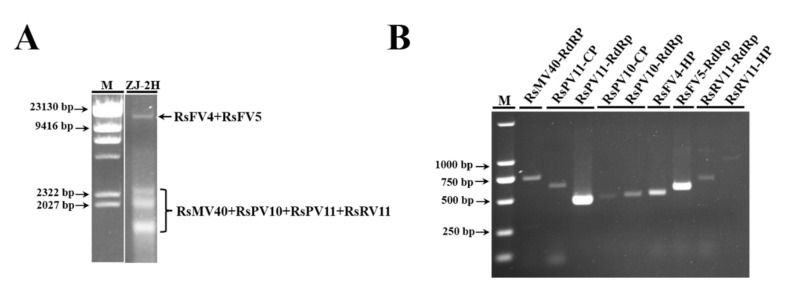
Identification of mycoviruses in *Rhizoctonia solani* AG-3 PT strain ZJ-2H. (**A**) Multiple bands of double-stranded RNA (dsRNA) extracted from *R. solani* AG-3 PT strain ZJ-2H were visualized by 1% agarose gel electrophoresis and subsequently purified. M: λ-*Hind* III digest; ZJ-2H: dsRNA extracted from *R. solani* AG-3 PT strain ZJ-2H. (**B**) Products of reverse transcription-polymerase chain reaction (RT-PCR) using viral-specific primers designed based on the nucleotide sequences of RNA dependent RNA polymerase (RdRp), coat protein (CP), or hypothetical protein (HP) in the six mycoviruses. Products were visualized and purified by 1% agarose gel electrophoresis. Total RNA was extracted from *R. solani* AG-3 PT strain ZJ-2H and used as an RNA template in RT-PCR. M: DNA molecular marker DL 2000.

Multiple bands of dsRNA extracted from *R. solani* AG-3 PT strain ZJ-2H ranging from approximately 1000 bp to 10,000 bp were visualized via electrophoresis (Figure 1A). Metatranscriptome sequencing on an Illumina HiSeq 2500 platform was conducted to characterize the mycovirome of *R. solani* AG-3 PT strain ZJ-2H. A total of 86,267,892 raw reads were obtained. After filtering, the resulting clean reads were de novo assembled into 27,781 contigs (Table S4) using CLC Genomics Workbench version 6.0.4 software. Results of BLASTx searches against the NCBI NR database revealed that the nine contigs represented six distinct mycoviruses co-infecting *R. solani* AG-3 PT strain ZJ-2H. Analysis of the putative amino acid (aa) sequences of the viral contigs indicated that the six mycoviruses belonged to the families *Mitoviridae* and *Partitiviridae*, and the proposed families Bipatitiviridae and Fusariviridae based on homology to previously sequenced mycoviruses. All of the mycoviral contigs with their identities, best matches, and names are listed in Table 1. The six mycoviruses identified from the metatranscriptome analysis were further confirmed to be present in strain ZJ-2H (Figure 1B) by RT-PCR assays using total RNA extracted from the strain ZJ-2H and viral-specific primers for each mycovirus (Table S1).

### 3.2. Genome Organization and Phylogenetic Analysis of Putative Members of the Proposed Family Fusariviridae

**Figure 2:**
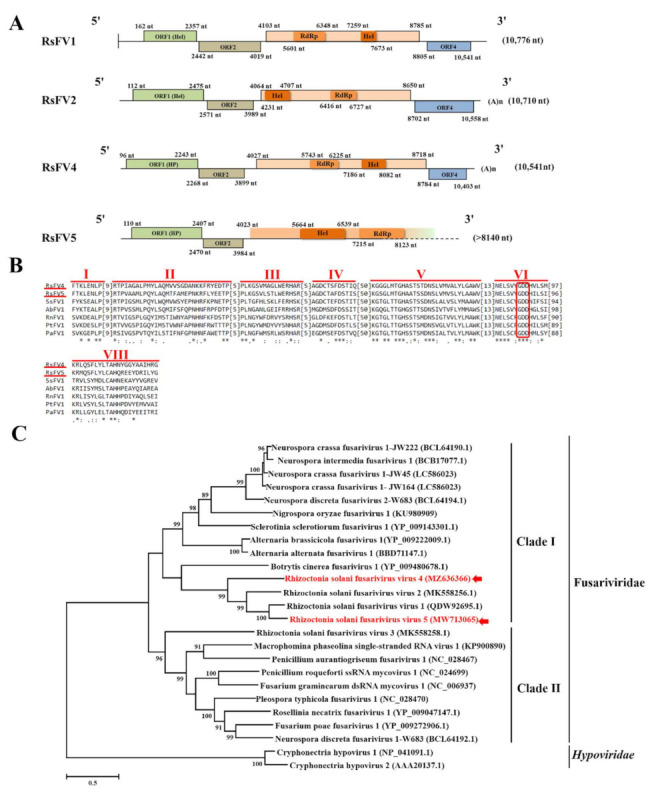
Genome organization, multiple alignments of amino acid (aa) sequences of RNA-dependent RNA polymerase (RdRp), and phylogenetic analysis of aa sequences of RdRp of Rhizoctonia solani fusarivirus 4 (RsFV4) and Rhizoctonia solani fusarivirus 5 (RsFV5). (**A**) Schematic diagrams represent the genome organization of RsFV4, RsFV5, Rhizoctonia solani fusarivirus 1 (RsFV1), and Rhizoctonia solani fusarivirus 2 (RsFV2), respectively. Each virus contains three or four open reading frames (ORFs). (**B**) Multiple aa sequence alignments of RdRp of RsFV4, RsFV5, and five other representative fusariviruses. Seven conserved motifs (I to VI, and VIII) are indicated. The highly conserved GDD tripeptide present in seven mycoviruses are indicated by red boxes. “*” indicates identical amino acids, “:” indicates high chemically similar amino acids, and “.” indicates low chemically similar amino acids. (**C**) Phylogenetic analysis of aa sequences of RdRp of RsFV4, RsFV5, 21 other representative fusariviruses, and two hypoviruses. Viruses in the proposed family Fusariviridae are clustered in two clades (Clade I and Clade II), and RsFV4 and RsFV5 together with RsFV1 and RsFV2 cluster together in Clade I. The names of reference and GenBank accession numbers of mycoviruses are listed in Table S5.

Two contigs (contig978 and contig6043) (Table 1) assembled from the metatranscriptome data were identified as two new members of the newly proposed +ssRNA virus family, Fusariviridae. The two new mycoviruses were named Rhizoctonia solani fusarivirus 4 (RsFV4) and Rhizoctonia solani fusarivirus 5 (RsFV5) to conform with the naming of previously reported Rhizoctonia solani fusarivirus 1 (RsFV1), Rhizoctonia solani fusarivirus 2 (RsFV2), and Rhizoctonia solani fusarivirus 3 (RsFV3) [35]. The whole genome sequence (10,541 nt in length) of RsFV4 and the partial genome sequence (8140 nt in length) of RsFV5 were obtained using the results of the RNA-Seq and terminal sequence data (Figure 2A). The whole and partial genome sequences for RsFV4 and RsFV5 were submitted to NCBI under the accession numbers MZ636366 and MW713065, respectively.

The aa sequences of RdRp of RsFV4 and RsFV5 share 49.21% identity and 71.34% identity with RsFV1 (accession number: QDW92695) and belong to the proposed family Fusariviridae (Table 1). There are also some differences in genome organization between the two mycoviruses (RsFV4 and RsFV5) and RsFV1 (Figure 2A). RsFV1 possesses four ORFs (ORFs 1, 2, 3, and 4). Among the four ORFs, ORF1 encodes helicase (Hel), ORF3 encodes RdRp and another Hel with the 5’-ORF encoding RdRp, and both ORF2 and ORF4 encode unknown proteins. RsFV4 also contains four ORFs with a polyadenylated region at the 3’-terminus, however, among the four ORFs, ORF1 encodes a hypothetical protein, ORF3 (the longest ORF) encodes a polyprotein with 1563 aa that includes RdRp and Hel with the 5’-ORF encoding RdRp, and both ORF2 and ORF4 encode unknown proteins. RsFV5 possesses ORF1, ORF2, and an incomplete ORF3. Among the three ORFs, ORF1 encodes a hypothetical protein, ORF2 encodes an unknown protein, and ORF3 encodes a polyprotein that includes RdRp and Hel with the 5’-ORF encoding Hel.

Seven conserved motifs (I–VI, and VIII) were identified in the aa sequence of RdRp encoded by ORF3 of RsFV4 and RsFV5, while no conserved motif was found in ORFs 1, 2, and 4 of RsFV4 or ORFs 1 and 2 of RsFV5. Furthermore, a GDD tripeptide (the hallmark of most viral RdRps) was found within the RdRp domain of RsFV4 and RsFV5 (Figure 2B).

Phylogenetic analysis indicated that the currently recognized fusariviruses within the proposed family Fusariviridae can be divided into two clades (Clade I and Clade II). RsFV4 and RsFV5, together with RsFV1 and RsFV2, from the proposed family Fusariviridae cluster in Clade I (Figure 2C). Although both RsFV4 and RsFV5 contain a single +ssRNA genome with about 10,000 nt in length, which is similar to members in the family *Hypoviridae*, the two fusariviruses and hypoviruses are distinctly separate (Figure 2C).

### 3.3. Genome Organization and Phylogenetic Analysis of the Putative Member of the Family Mitoviridae

**Figure 3:**
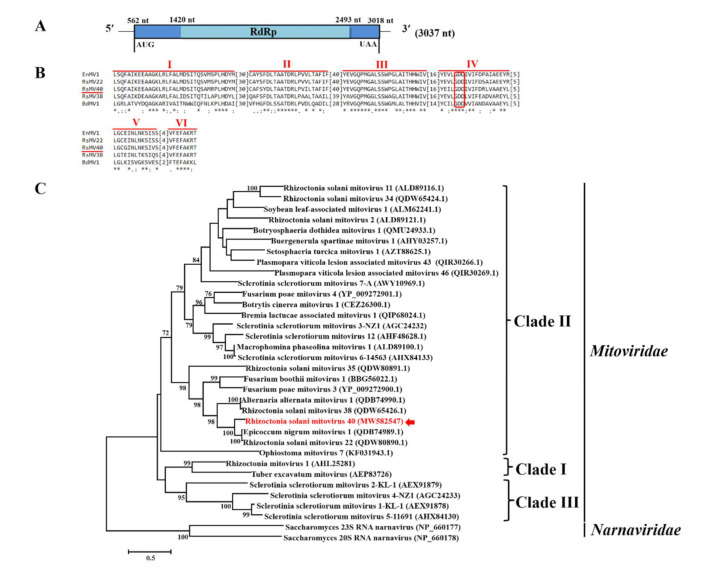
Genome organization, multiple alignments of amino acid sequences of RNA-dependent RNA polymerase (RdRp), and phylogenetic analysis of amino acid sequences of RdRp of Rhizoctonia solani mitovirus 40 (RsMV40). (**A**) Schematic diagram illustrating the genome organization of RsMV40. RsMV40 contains a single open reading frame (ORF) encoding RdRp, which is initiated by an AUG codon and is terminated by a UAA codon. (**B**) Multiple alignment of aa sequences of RdRp of RsMV40 and four representative mitoviruses. The presence of six conserved motifs (I to VI) is shown. The highly conserved GDD tripeptide present in the five mycoviruses are indicated by red boxes. “*” indicates identical amino acids, “:” indicates high chemically similar amino acids, and “.” indicates low chemically similar amino acids. (**C**) Phylogenetic analysis of aa sequences of RdRp of RsMV40 and 31 other representative members of the family *Mitoviridae* and two narnaviruses. Viruses in the family *Mitoviridae* were clustered in three clades (Clade I, Clade II, and Clade III), and RsMV40 (red arrow) clustered in Clade II. The names and GenBank accession numbers of the reference mycoviruses are listed in Table S5.

One contig (First_contig5) (Table 1) assembled from the metatranscriptome sequencing data was 2806 nt in length and phylogenetically related to members in the +ssRNA virus family, *Mitoviridae*. After obtaining the terminal sequences, the whole genome of this mycovirus was assembled and found to be 3037 nt in length (Figure 3A), sharing 73.97% identity with the RdRp of Epicoccum nigrum mitovirus 1 (EnMV1) (accession number: QDB74989). This virus was designated as Rhizoctonia solani mitovirus 40 (RsMV40) and identified as a new member of the family *Mitoviridae* based on species demarcation criteria previously designated by the International Committee on Taxonomy of Viruses (ICTV, July 2021) (https://talk.ictvonline.org/taxonomy/ (accessed on 1 April 2022)). Analysis of genome organization using both mitochondrial codon usage and standard codon usage revealed that RsMV40 possessed a single integral ORF encoding RdRp, which is initiated by the start codon AUG and terminated by the stop codon UAA (Figure 3A). The whole genome sequence of RsMV40 was deposited in GenBank under accession number MW582547.

Multiple alignments identified six conserved motifs (I–VI) containing the GDD tripeptide (characteristics of RdRp in mitoviruses) present in the aa sequence of RdRp in RsMV40 (Figure 3B). Results of the phylogenetic tree constructed based on the aa sequence of RdRp in RsMV40 and representative members of the genus *Mitovirus* indicated that RsMV40 and the other mitoviruses identified in *R. solani*, such as Rhizoctonia solani mitovirus 2 (RsMV2), Rhizoctonia solani mitovirus 11 (RsMV11), Rhizoctonia solani mitovirus 22 (RsMV22), Rhizoctonia solani mitovirus 34 (RsMV34), and Rhizoctonia solani mitovirus 35 (RsMV35), cluster together in Clade II (Figure 3C).

### 3.4. Genome Organization and Phylogenetic Analysis of Partiti-Like Viruses

Six contigs (contig315, contig644, contig1221, contig3112, contig11433, and contig14233) assembled from the metatranscriptome sequencing data shared varying degrees of homology with partiti-like viruses in the NCBI NR database, as revealed by BLASTx analysis. The six contigs were further characterized (Table 1).

Contig315 and contig644 encode a CP and RdRp, respectively, sharing affinity with the best BLAST hit of the aa sequence of CP (24.49% identity) and RdRp (55.65% identity) of the alphapartitivirus, Ceratobasidium partitivirus (accession numbers: AOX47570 for RdRp, AOX47601.1 for CP). Contig1221 and contig3112 encode CP and RdRp, respectively, which were most closely related to the aa sequences of CP (29.44% identity) and RdRp (61.33% identity) of the alphapartitivirus, White clover cryptic virus 1 (accession numbers: YP_086754 for RdRp, YP_086755 for CP). Thus, contig1221/3112 and contig315/644 were designated as partial sequences of two new species of the genus *Alphapartitivirus* and named Rhizoctonia solani partitivirus 10 (RsPV10) and Rhizoctonia solani partitivirus 11 (RsPV11), respectively. Both the 5’- and 3’-UTRs of RsPV10 and RsPV11 were conserved (Figure S1).

**Figure 4:**
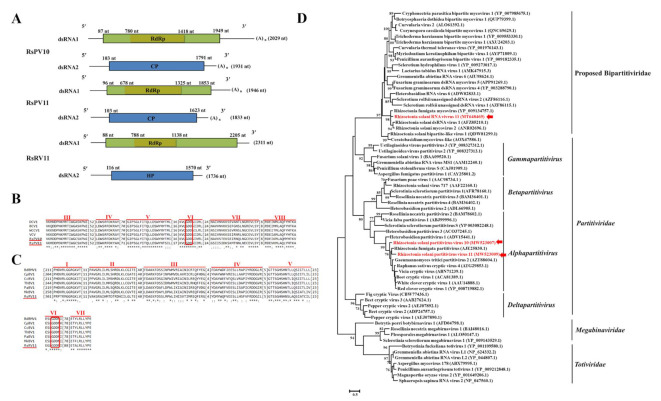
Genome organization, multiple alignments of amino acid (aa) sequence of RNA-dependent RNA polymerase (RdRp), and phylogenetic analysis of aa sequences of RdRp of Rhizoctonia solani partitivirus 10 (RsPV10), Rhizoctonia solani partitivirus 11 (RsPV11), and Rhizoctonia solani RNA virus 11 (RsRV11). (**A**) Schematic diagrams illustrating the genome organization of RsPV10, RsPV11, and RsRV11. Each of the three mycoviruses possess two double-stranded RNAs (dsRNAs), namely dsRNA1 (the longer dsRNA) and dsRNA2 (the shorter dsRNA). The dsRNA1 and dsRNA2 in RsPV10 and RsPV11 encode RdRp and coat protein (CP), respectively, while the dsRNA1 and dsRNA2 in RsRV11 encode RdRp and a hypothetical protein (HP), respectively. The 3’-terminus of dsRNA1 and dsRNA2 in RsPV10 and RsPV11 possess a poly adenylate (polyA) sequence, which is not found in the 3’-terminus of either dsRNA1 or dsRNA2 of RsRV11. (**B**) Multiple aa alignments of RdRp in RsPV10, RsPV11, and four other representative partitiviruses in which six conserved motifs (III to VIII) are indicated. (**C**) Multiple aa alignments of RdRp in RsRV11 and six other representative proposed bipartitiviruses in which seven conserved motifs (I to VII) are indicated. The highly conserved GDD tripeptide is present in partitiviruses and bipartitiviruses and is indicated by red boxes. “*” indicates identical amino acids, “:” indicates high chemically similar amino acids, and “.” indicates low chemically similar amino acids. (**D**) Phylogenetic analysis of aa sequences of RdRp in RsPV10, RsPV11, RsRV11, and 29 representative members of *Partitiviridae*, seven members of *Totiviridae*, and four members of *Megabirnaviridae* as well as 22 members of proposed family, Bipartitiviridae. The dendrogram indicates that RsPV10 and RsPV11 cluster in the genus *Alphapartitivirus*, while RsRV11 clusters in the proposed family, Bipartitiviridae. The names and GenBank accession numbers of reference mycoviruses are listed at Table S5.

Analysis of whole genome organization revealed that both RsPV10 and RsPV11 possess two dsRNA segments, encoding RdRp and CP (Figure 4A). The longer dsRNA (dsRNA1) of RsPV10 and RsPV11 encode an RdRp of 2029 bp and 1946 bp in length and a calculated molecular mass of 72.99 kDa and 69.31 kDa, respectively. The shorter dsRNA (dsRNA2) of RsPV10 and RsPV11 encode a CP of 1931 bp and 1833 bp in length and a calculated molecular mass of 55.68 kDa and 55.42 kDa, respectively. All four dsRNAs possess a polyadenylated region at the 3’-terminus. The whole genome sequences of RsPV10 and RsPV11 were deposited in GenBank under the accession numbers MW523007 (for dsRNA1 of RsPV10), MZ467297 (for dsRNA2 of RsPV10), MW523009 (for dsRNA1 of RsPV11), and MW523010 (for dsRNA2 of RsPV11).

Contig11433 and contig14233 shared the highest homology with the aa sequences of HP (56.13%) and RdRp (72.40%) of Rhizoctonia fumigata mycovirus (RfMV) (accession numbers: YP_009134757.1 for RdRp, YP_009134758.1 for HP), respectively, followed by the aa sequences of HP (42.63%) and RdRp (54.53%) of Rhizoctonia solani dsRNA virus 1 (RsRV1) (accession numbers: QXI69642.1 for RdRp, AFZ85211.1 for HP). Therefore, contig11433 and contig14233 were designated as partial sequences of a new mycovirus named Rhizoctonia solani RNA virus 11 (RsRV11). Whole genome analysis revealed that RsRV11 contained two dsRNAs (dsRNA1 and dsRNA2) that were 2311 bp and 1736 bp in length, respectively. The 5’- and 3’-UTRs of the two dsRNAs of RsRV11 were conserved. Additionally, dsRNA1 possesses a single ORF encoding an RdRp with a molecular mass of 80.38 kDa, while dsRNA2 possesses a single ORF encoding HP (calculated molecular mass of 54.13 kDa) with unknown function. These two dsRNAs did not possess a polyadenylated region at the 3’-terminus. The whole genome sequences of dsRNA1 and dsRNA2 of RsRV11 were deposited in GenBank under the accession numbers MT648469 and MT648470, respectively.

Six conserved motifs (motif III-VIII) with a GDD tripeptide (characteristic of the RdRp of alphapartitiviruses) within motif VI were identified in RsPV10 and RsPV11 (Figure 4B), while seven conserved motifs (motif I-VII) with a GDD tripeptide within motif VI (Figure 4C) were found in RsRV11, as well as in six representative members (BdBMV1, CpBV1, CcBV1, ThBV1, PaBV1, and MkBV1) of the proposed family, Bipartitiviridae. Phylogenetic analysis of the RdRp aa sequences of these three partiti-like viruses revealed that RsPV10 and RsPV11 cluster with members of the genus *Alphapartitivirus*, while RsRV11 clusters with other previously reported unclassified mycoviruses. Thus, RsRV11 was presumed to be distinct from the families *Partitviridae*, *Megabirnaviridae*, and *Totiviridae* (Figure 4D). RsRV11 and other homologous mycoviruses have been proposed to represent a new family, namely Bipartitiviridae [35].

### 3.5. Analysis of the Secondary Structures of the Termini of Mycoviruses

**Figure 5:**
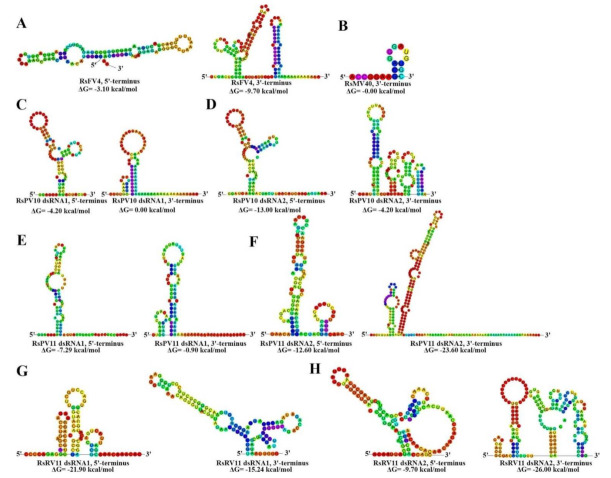
Predicted secondary structure of the 5’− and 3’−untranslated regions (UTRs) of the five mycoviruses with a complete genome sequence identified in *Rhizoctonia solani* AG-3 PT strain ZJ-2H. (**A**) Predicted secondary structure of the 5’− and 3’−UTRs of Rhizoctonia solani fusarivirus 4 (RsFV4). (**B**) Predicted secondary structure of the 3’−UTR of Rhizoctonia solani mitovirus 40 (RsMV40). (**C**) Predicted secondary structure of the 5’− and 3’−UTRs of double-stranded RNA 1 (dsRNA1) of Rhizoctonia solani partitivirus 10 (RsPV10). (**D**) Predicted secondary structure of the 5’− and 3’−UTRs of dsRNA2 of RsPV10. (**E**) Predicted secondary structure of the 5’− and 3’−UTRs of dsRNA1 of Rhizoctonia solani partitivirus 11 (RsPV11). (**F**) Predicted secondary structure of the 5’− and 3’−UTRs of dsRNA2 of RsPV11. (**G**) Predicted secondary structure of the 5’− and 3’−UTRs of dsRNA1 of Rhizoctonia solani RNA virus 11 (RsRV11). (**H**) Predicted secondary structure of the 5’− and 3’−UTRs of dsRNA2 of RsRV11. The RNAs were folded, and the free energy (ΔG) was estimated using the RNAfold web server (http://rna.tbi.univie.ac.at/cgi-bin/RNAWebSuite/RNAfold.cgi (accessed on 19 September 2021)).

Analysis of the 5’- and 3’-UTRs of the five mycoviruses (RsFV4, RsMV40, RsPV10, RsPV11, and RsRV11) with a complete genome was conducted. Among the five viruses, the 5’-UTR (561 nt in length) of RsMV40 is the longest, and longer than most representative members of the family *Mitoviridae* [44,45,46,47]. The dsRNA2 of RsPV11 has the longest 3’-UTR (210 nt in length), while the 3’-UTR of RsMV40 is the shortest (19 nt in length), and much shorter than most other mitoviruses [46] (Figure 2A, Figure 3A, Figure 4A). Except for RsMV40 and the dsRNA1 of RsPV11, whose 5’-UTR is longer than their own 3’-UTR, the length of the 5’-UTR of the mycoviral segments from the other three mycoviruses (RsFV4, RsPV10, and RsRV11) and the dsRNA2 of RsPV11 is shorter than that of their own 3’-UTR (Figure 3A and Figure 4A). The predicted terminal structures of the five novel mycoviruses are presented in Figure 5 and Figure S2. The 5’-UTR of RsFV4 (Figure 5A) and the 3’-UTR of RsMV40 (Figure 5B) are predicted to form a hairpin structure, while the 5’- and 3’-UTRs of RsPV10, RsPV11, and RsRV11 are predicted to form stem–loop structures without a pseudoknot structure. The 5’-UTR of dsRNA1 and dsRNA2 of RsPV10 were also highly conserved, forming extremely similar secondary structures (Figure 5C,D).

### 3.6. Observations of Virus Particles and Confirmation of the Mycoviruses Contributing to the Assembly of Virus Particles

Virus particles extracted from *R. solani* AG-3 PT strain ZJ-2H were spherical, and approximately 30 nm in diameter (Figure S3A). RT-PCR products amplified from the RNA of the virus particles using viral specific primers (Table S1) were sequenced. As a result, RsPV10 and RsPV11 were confirmed to be the mycoviruses present in and responsible for assembling the virus particles extracted from strain ZJ-2H, while sequence evidence for the four other mycoviruses (RsFV4, RsFV5, RsMV40, and RsRV11) was not obtained from the virus particles (Figure S3B).

## 4. Discussion

In the present study, metatranscriptome sequencing was used to characterize the mycovirome in *R. solani* AG-3 PT strain ZJ-2H. The metatranscriptome sequencing data and BLASTx queries were used to identify nine contigs representing six novel mycoviruses in strain ZJ-2H. The whole genome sequence of five of the mycoviruses (RsFV4, RsMV40, RsPV10, RsPV11 and RsRV11) were assembled from sequences obtained by RNA sequencing (RNA-Seq) combined with terminal sequences obtained using RACE methodology. We failed, however, to determine the complete sequence of RsFV5, which may have been due to the specialized structure of the 3’-terminus in this mycovirus. Three new species of mycoviruses, RsPV10 (dsRNA, family *Partitiviridae*, genus *Alphapartitivirus*), RsPV11 (dsRNA, family *Partitiviridae*, genus *Alphapartitivirus*), and RsMV40 (+ssRNA, family *Mitoviridae*, geus *Mitovirus*) were identified based on the classification criteria designated by the ICTV. While species demarcation criteria have not been designated for members of the proposed families Fusariviridae and Bipartitiviridae, RsFV4 (+ssRNA, proposed family Fusariviridae), RsFV5 (+ssRNA, proposed family Fusariviridae), and RsRV11 (dsRNA, proposed family Bipartitiviridae) were designated as new species in their corresponding families based on their low aa identity to currently certified viruses (Table 1). To the best of our knowledge, this is the first report of six novel mycoviruses containing +ssRNA and dsRNA genomes that co-infect a single strain within the *R. solani* AG-3 PT.

Mycovirus co-infections have been reported to occur in diverse fungi [9], and may not be due to horizontal transmission among homologous fungal hosts, but rather associated with the phylogenetic evolution of diverse co-infecting mycoviruses [48,49,50]. An increasing number of metagenomic evolutionary studies have indicated that there are many strong evolutionary connections among RNA viruses that have been previously thought to be unrelated, especially for +ssRNA and dsRNA viruses. Iterative computational procedures have indicated that diverse dsRNA viruses may descend from +ssRNA viruses [51], a premise that has been laterally confirmed by the fact that +ssRNA and dsRNA viruses always co-infect the same host [46,49]. A unique intimate relationship between a +ssRNA virus, yado-kari virus (YkV1), and a dsRNA virus, yado-nushi virus (YnV1), co-infecting a single hypovirulent strain has also been demonstrated, in which YkV1 highjacks the capsid of YnV1 for trans-encapsidation of YkV1 RNA and RdRp [52,53]. Our study revealed that three +ssRNA mycoviruses (belonging to the proposed family Fusariviridae and the family *Mitoviridae*) along with three dsRNA viruses (belonging to the family *Partitiviridae* and the proposed family Bipartitiviridae) co-infect a single strain of *R. solani* AG-3 PT. This finding provides new insights into the evolutionary provenance of +ssRNA and dsRNA mycoviruses.

The family Fusariviridae was originally proposed by Zhang et al. [54] in 2014, and included two members, Fusarium graminearum virus 1 (FgV1) [55] and Rosellinia necatrix fusarivirus 1 (RnFV1) [54]. Since then, several fusariviruses have been identified in *Neurospora* [56], *Fusarium* [54,56,57], *Rosellinia* [54], and *Rhizoctonia* [35]. Members of the proposed family Fusariviridae were initially characterized as possessing one +ssRNA genome, encoding two ORFs, with the 5’-ORF encoding an RdRp [54,55,56]. Both RsFV1 and RsFV2, the two fusariviruses found in *Rhizoctonia*, have four ORFs. ORF1 encodes Hel, ORF3 encodes RdRp and another Hel [35], and both ORF2 and ORF4 encode unknown proteins. The 5’-ORF3 of RsFV1 encodes RdRp, while the 5’-ORF3 of RsFV2 encodes Hel. RsFV4, identified in the present study, also contains four ORFs. ORF1 encodes a hypothetical protein, ORF3 encodes an RdRp and a Hel with the 5’-ORF3 encoding an RdRp, and both ORF2 and ORF4 encode unknown proteins. The partial genome of RsFV5 obtained in the present study possesses three discontinuous ORFs. ORF1 encodes a hypothetical protein, ORF2 encodes an unknown protein, and ORF3 encodes a Hel and an RdRp with the 5’-ORF encoding a Hel. Overlapping discontinuous regions of RsFV4 and RsFV5 were RT-PCR amplified using the viral-specific primers listed in Table S2 and the resulting PCR products were sequenced to confirm that the discontinuous ORFs contained in RsFV4 and RsFV5 were precise. Based on our obtained data, we propose that fusariviruses may contain two to four ORFs, with the longest ORF encoding RdRp and Hel, with the first ORF of fusariviruses not always encoding Hel.

Results of the phylogenetic analysis conducted in the present study indicated that fusariviruses cluster in two clades (Clade I and Clade II), which is in agreement with the dendrogram reported in Honda et al. [56]. RsFV4 and RsFV5 clustered in Clade I. Although FgV1 and RnFV1 (the only two well-studied representative fusariviruses) clustered in the same clade (Clade II) in the phylogenetic tree (Figure 2C), the genome organization and gene expression strategy in these two fusariviruses are distinctly different. FgV1 possesses four ORFs, with the last three ORFs being expressed through sub-genomic RNAs [55,57,58], while RnFV1 only possesses two ORFs, with the downstream ORF being expressed via an unknown mechanism [55]. Fusariviruses isolated from *R. solani* also clustered in the same clade (Clade I), although their genomic organization was not entirely similar. Based on the collective data, we presume that fusariviruses may evolve together with their fungal hosts, and that divergence in their nucleotide sequences may occur. Since it is expected that an increasing number of fusariviruses will be identified, further classification of members within the proposed family Fusariviridae will require additional research on the evolutionary processes in fusariviruses.

*Mitoviridae* is a newly established family designated by the ICTV (2019), comprising mitoviruses from plants and fungi that typically replicate and persist in the mitochondrion of a host [11,59]. The numerous mitoviruses identified in *Rhizoctonia* have large 5’-UTRs, such as Rhizoctonia solani mitovirus 4 (RsMV4, 869 nt), Rhizoctonia solani mitovirus 21 (RsMV21, 940 nt), and Rhizoctonia solani mitovirus 35 (RsMV35, 936 nt), suggesting that a large 5’-UTR may be a common feature of mitoviruses associated with *Rhizoctonia*. In the present study, RsMV40, a new member of the family *Mitoviridae*, was shown to possess +ssRNA as the genome encoding RdRp. The 5’-UTR of RsMV40 is 561 nt in length, which is longer than that of almost all other mitoviruses except Cryphonectria cubensis mitovirus 2a (CcMV2a, 892 nt) and Tuber excavatum mitovirus (TeMV, 793 nt) [47], which have been isolated from *Cryphonectria cubensis* and *Tuber excavatum*, respectively. In contrast, the 3’-UTR of RsMV40 is only 20 nt long, which is shorter than those of other previously reported mitoviruses [46]. Based on previous reports, most mitoviruses typically contain 7-12 UGA codons that encode tryptophan rather than acting as a stop codon [60,61]. RsMV40, however, possesses a UAA codon at 3016 nt that functions as a stop codon rather than a UGA codon. In some cases, when a UGA codon is not present in mitoviruses, tryptophan is encoded by a UGG codon, which does not alter transcription for either the mitochondrial or nuclear genetic code, such as RsMV40 in the present study, which possesses 14 UGG codons encoding tryptophan. Since UGA (Trp) is a rare mitochondrial codon in fungi [62], fungal mitoviruses, especially mitoviruses associated with *Rhizoctonia* [12,32,62,63], are often translated using nuclear genetic codes.

The phylogenetic analysis conducted in our study indicated that mitoviruses clustered into three clades (Clade I, Clade II, and Clade III), which is consistent with the previous reports of Xu et al. [44] and Mizutani et al. [45]. RsMV40 clustered in Clade II together with other representative mitoviruses found in *R. solani*, such as RsMV2, RsMV11, RsMV22, RsMV34, and RsMV35 (Figure 3C). According to previous reports, mitoviruses in the same fungal species (such as *Sclerotinia sclerotiorum* and *Ophiostoma novo-ulmi*) cluster randomly into different groups; however, some mitoviruses found in different fungal species exhibit a relatively close relationship [44,64,65,66]. It is assumed from these reports that some mitoviruses may have existed before their fungal hosts diverged [67,68]. Mitoviruses found in *R. solani* may have evolved together with the DNA genomes of their fungal hosts. The long-term co-evolution of mitoviruses with their host *Rhizoctonia,* however, may not have induced major sequence divergence in their mitoviral genomes.

Recently, an increasing number of unclassified partitiviruses have been identified in diverse fungi belonging to the newly proposed genera, Zetapartitivirus (such as Aspergillus flavus partitivirus 1 [69] and Colletotrichum acutatum RNA virus 1 [70]) and Epslonpartitivirus (such as Hubei partiti-like virus 10 [7] and Penicillium aurantiogriseum partiti-like virus [71]), rather than other recognized genera, such as *Alphapartitivirus*, *Betapartitivirus*, *Gammaparititvirus*, or *Deltapartitivirus* [72]. In the current study, three bipartite dsRNA mycoviruses, RsPV10, RsPV11, and RsRV11, were identified in *R. solani* AG-3 PT strain ZJ-2H. Among them, RsPV10 and RsPV11 were identified as two new members of the genus *Alphapartitivirus* and confirmed to assemble into virus particles in strain ZJ-2H. Although the genome of RsRV11 contains two dsRNAs, it has a distinct relationship with RsPV10 or RsPV11, and clusters in an independent branch (Figure 4D), hypothetically named as the proposed family Bipartitiviridae [35,73]. Notably, RsRV11 shares the highest nucleotide sequence identity with RfMV, which was previously identified as a member of the family *Megabirnaviridae* [74]. Based on the phylogenetic tree constructed in our present study (Figure 4D) as well as several recent reports [37,75], RsRV11 together with the RfMV cluster in the proposed family Bipartitiviridae, indicating that RfMV has a distinct relationship with members of the family *Megabirnaviridae*. In fact, RfMV contains one large dsRNA (9907 nt) as a genome encoding two proteins, which is different from the genomes of representative members within the family *Megabirnaviridae* (containing two large dsRNAs as a genome) [76,77,78] or the proposed family Bipartitiviridae (containing two dsRNAs with approximate 2000 nt in length) [79,80]. Thus, further studies on the evolutionary relationship between the family *Megabirnaviridae* and the proposed family Bipartitiviridae should be considered in the future.

The newly proposed family Bipartitiviridae encompasses diverse mycoviruses having a non-uniform genome organization. In general, bipartitiviruses, such as Rhizoctonia solani dsRNA virus 1 (RsRV1) [71] and RsRV11 in the present study, possess two dsRNA segments, dsRNA1 and dsRNA2, that encode RdRp and a hypothetical protein, respectively. Sometimes, the dsRNA2 in bipartitiviruses contains two ORFs encoding two unknown proteins, such as Fusarium graminearum virus 4 (FgV4) [81]. Importantly, dsRNA2 is undetectable in the two bipartitiviruses, Gremmeniella abietina RNA Virus 6 (GaRV6) and Heterobasidion RNA virus 6 (HeRV6) [82]. Therefore, the diverse genomic organization present in bipartitiviruses suggests that the classification of members within the proposed Bipartitiviridae will be complex. Further identification of new members of this proposed family are needed to promote the establishment of the family Bipartitiviridae and fully characterize its features.

The evolutionary provenance of RNA viruses appears to be complex and pluralistic [83,84,85]. The analysis of the genomic information generated in the present study may provide evidence of the evolutionary relationship between +ssRNA and dsRNA viruses. Moreover, our study may offer a foundation to explore mycovirus interaction systems and the evolutionary relationship between multiple mycoviruses with different genome types and their fungal hosts. Therefore, the interactions between these six mycoviruses and their influence on their host fungus, *R. solani* AG-3 PT strain ZJ-2H, need to be further investigated.

## Figures and Tables

**Table 1:** Six novel mycoviruses identified in *Rhizoctonia solani* AG-3 PT strain ZJ-2H obtained through metatranscriptome sequencing and rapid amplification of cDNA ends (RACE).

Virus Name	Accession Number	Contig ID	Contig Length (nt)	Full Length (nt)	BLASTx First Hit	Identity	Query Cover
Rhizoctonia solani fusarivirus 4 (RsFV4)	MZ636366	contig978	10,416	10,541	Rhizoctonia solani fusarivirus 1	49.21%	32%
Rhizoctonia solani fusarivirus 5 (RsFV5)	MW713065	contig6043	8134	>8140	Rhizoctonia solani fusarivirus 1	71.34%	49%
Rhizoctonia solani mitovirus 40 (RsMV40)	MW582547	First_contig5	2806	3037	Epicoccum nigrum mitovirus 1	73.97%	81%
Rhizoctonia solani partitivirus 10 (RsPV10)	MW523007	contig3112	2001	2029	White clover cryptic virus 1	61.33%	86%
	MZ467297	contig1221	1700	1713	White clover cryptic virus 1	29.44%	30%
Rhizoctonia solani partitivirus 11 (RsPV11)	MW523009	contig644	1921	1946	Ceratobasidium partitivirus	55.65%	89%
	MW523010	contig315	1788	1833	Ceratobasidium partitivirus	24.49%	82%
Rhizoctonia solani RNA virus 11 (RsRV11)	MT648469	contig14243	1730	1736	Rhizoctonia fumigata mycovirus	72.40%	91%
	MT648470	contig11433	2300	2311	Rhizoctonia fumigata mycovirus	56.13%	79%

## Data Availability

The sequences reported in the present manuscript have been deposited in the GenBank database under accession numbers MZ636366, MW713065, MW582547, MW523007, MZ467297, MW523009, MW523010, MT648469, and MT648470.

## Supplementary Materials

The following are available online at https://www.mdpi.com/article/10.3390/v14040813/s1, Figure S1: Sequence alignments of 5’- and 3’-untranslated regions (UTRs) of dsRNA1 and dsRNA2 of Rhizoctonia solani partitivirus 10 (RsPV10) (**A**) and Rhizoctonia solani partitivirus 11 (RsPV11) (**B**). Identical nucleotides are indicated by asterisks, and conserved purines or pyrimidines are indicated by dots. Figure S2: Secondary structure of the 5’-untranslated region (UTR) of Rhizoctonia solani mitovirus 40 (RsMV40). The schematic diagram illustrates the multiple stem loops and panhandle structure of the 5’-UTR of RsMV40. The RNAs were folded, and the free energy (ΔG) was estimated using RNAfold web server (http://rna.tbi.univie.ac.at/cgi-bin/RNAWebSuite/RNAfold.cgi (accessed on 19 September 2021)). Figure S3: Observations of virus particles extracted from *Rhizoctonia solani* AG-3 PT strain ZJ-2H and confirmation of the mycoviruses contributing to the assembly of virus particles. (**A**) Transmission electron micrograph of virus particles extracted from *R. solani* AG-3 PT strain ZJ-2H. Red arrows indicate spherical virus particles, approximately 30 nm in diameter. (**B**) Products amplified by reverse transcription-polymerase chain reaction (RT-PCR) using viral-specific primers designed based on nucleotide sequences of RNA-dependent RNA polymerase (RdRp), coat protein (CP), and hypothetical protein (HP) and visualized using 1% agarose gel electrophoresis analysis to confirm the mycoviruses contributing to the assembly of virus particles. The total RNA of virus particles was extracted from *R. solani* AG-3 PT strain ZJ-2H and used as an RNA template. M: DNA molecular marker DL 2000; RsPV11-CP: Products amplified by CP primers in RsPV11; RsPV11-RdRp: Products amplified by RdRp primers in RsPV11; RsPV10-CP: Products amplified by CP primers in RsPV10; RsPV10-RdRp: Products amplified by RdRp primers in RsPV10; RsMV40-RdRp: Products amplified by RdRp primers in RsMV40; RsFV4-HP: Products amplified byHP primers in RsFV4; RsFV5-RdRp: Products amplified by RdRp primers in RsFV5; RsRV11-HP: Products amplified from hypothetical protein primers in RsRV11; RsRV11-RdRp: Products amplified from RdRp primers in RsRV11. Table S1: Primer pairs designed based on the nucleotide sequences of RNA dependent RNA polymerase (RdRp), coat protein (CP), or hypothetical protein (HP) in the six mycoviruses (Rhizoctonia solani mitovirus 40, Rhizoctonia solani fusarivirus 4, Rhizoctonia solani fusarivirus 5, Rhizoctonia solani parititivirus 10, Rhizoctonia solani parititivirus 11, and Rhizoctonia solani RNA virus 11). The primer pairs were used to confirm the presence of viral sequences in *Rhizoctonia solani* AG-3 PT strain ZJ-2H. Table S2: Primers used to validate the nucleotide sequences in the six mycoviruses (Rhizoctonia solani mitovirus 40, Rhizoctonia solani fusarivirus 4, Rhizoctonia solani fusarivirus 5, Rhizoctonia solani parititivirus 10, Rhizoctonia solani parititivirus 11, and Rhizoctonia solani RNA virus 11) based on sequences obtained through the metatranscriptome analysis of *Rhizoctonia solani* AG-3 PT strain ZJ-2H. Table S3: Primers used to determine the terminal sequences of the six mycoviruses (Rhizoctonia solani mitovirus 40, Rhizoctonia solani fusarivirus 4, Rhizoctonia solani fusarivirus 5, Rhizoctonia solani parititivirus 10, Rhizoctonia solani parititivirus 11, and Rhizoctonia solani RNA virus 11) associated with *Rhizoctonia solani* AG-3 PT strain ZJ-2H by rapid amplification of cDNA ends (RACE). Table S4: Clean reads selected after filtering the metatranscriptome sequences obtained from *Rhizoctonia solani* AG-3 PT strain ZJ-2H. Table S5: The reference viruses of families *Hypoviridae*, *Mitoviridae*, *Narnaviridae*, *Partitiviridae*, *Megabirnaviridae*, and *Totiviridae*, and proposed families Fusariviridae and Bipartitiviridae, which were retrieved from GenBank and used to conduct phylogenetic analysis.
