# Supplementary material for: Six Novel Mycoviruses Containing Positive Single-Stranded RNA and Double-Stranded RNA Genomes Co-Infect a Single Strain of the Rhizoctonia solani AG-3 PT
Source: Viruses. 2022 Apr 14;14(4):813. doi: 10.3390/v14040813 (PMC9025235; doi:10.3390/v14040813)
Supplement: Supplementary file 1 [file viruses-14-00813-s001.zip › Figure S1.pdf]

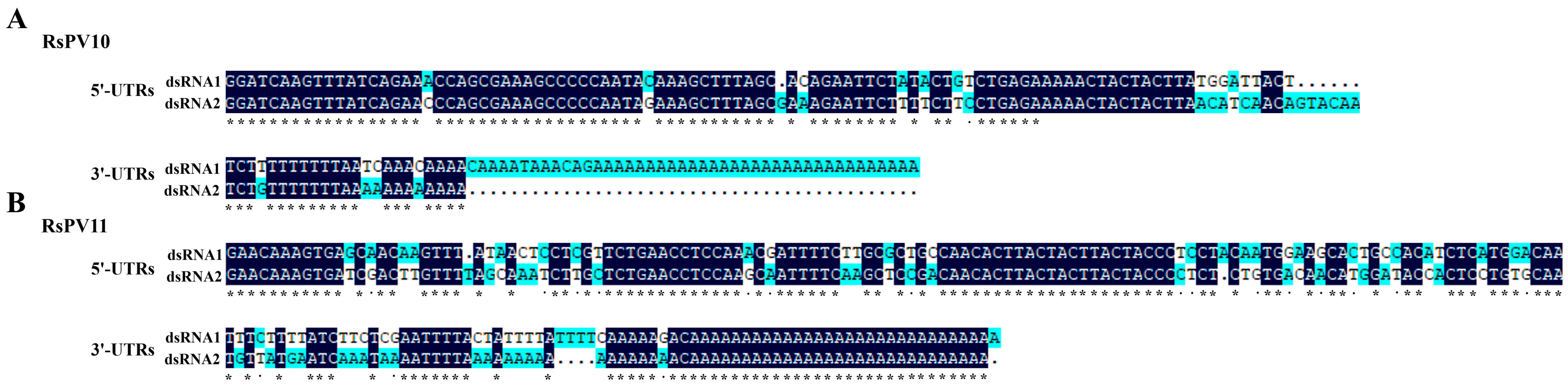
A
RsPV10
5'-UTRs
dsRNA1
dsRNA2
3'-UTRs
dsRNA1
dsRNA2
B
RsPV11
5'-UTRs
dsRNA1
dsRNA2
3'-UTRs
dsRNA1
dsRNA2
