# Supplementary material for: Six Novel Mycoviruses Containing Positive Single-Stranded RNA and Double-Stranded RNA Genomes Co-Infect a Single Strain of the Rhizoctonia solani AG-3 PT
Source: Viruses. 2022 Apr 14;14(4):813. doi: 10.3390/v14040813 (PMC9025235; doi:10.3390/v14040813)
Supplement: Supplementary file 1 [file viruses-14-00813-s001.zip › Table S1.pdf]

Table S1. Primer pairs designed on the basis of the nucleotide sequences of RNA dependent RNA polymerase (RdRp), coat protein (CP), or hypothetical protein (HP) in the six mycoviruses (Rhizoctonia solani mitovirus 40, Rhizoctonia solani fusarivirus 4, Rhizoctonia solani fusarivirus 5, Rhizoctonia solani partitivirus 10, Rhizoctonia solani partitivirus 11, and Rhizoctonia solani RNA virus 11). The primer pairs were used to confirm the presence of viral sequences in *Rhizoctonia solani* AG-3 PT strain ZJ-2H.

Primer Name	Sequence (5'-3')	Confirmed Viruses	Contig ID	Target Gene	Fragment Size (nt)	Annealing Temperature (°C)
RsMV40-F	TACGGGATAAGAAGAACGAACAGC	Rhizoctonia solani mitovirus 40 (RsMV40)	First_contig5	RdRp	768	56
RsMV40-R	AAGACCAAGACCAGGCCCAAT					54
RsFV4-F	GCACAAAACGCTGCCCAATACT	Rhizoctonia solani fusarivirus 4 (RsFV4)	contig978	HP	527	55
RsFV4-R	CTGCACGCGATAGGATGCATGT					57
RsFV5-F	CAATGGTCTGTGGGCTGTTGG	Rhizoctonia solani fusarivirus 5 (RsFV5)	contig6043	RdRp	669	56
RsFV5-R	ATTGGAGGTACGTCCGGTTCG					56
RsPV10-Rd-F	ATCGTCACCTTGGACTTTG	Rhizoctonia solani partitivirus 10 (RsPV10)	contig3112	RdRp	504	49
RsPV10-Rd-R	GAATACATCGCCTGGGTAA					49
RsPV10-CP-F	CATAAGGAACTTGGCTGTCTG		contig1221	CP	516	52
RsPV10-CP-R	ACAAAGAATGCGGAACTCTC					50
RsPV11-Rd-F	GGAAAGTATCATCGTACCATCCCTCG	Rhizoctonia solani partitivirus 11 (RsPV11)	contig644	RdRp	501	60
RsPV11-Rd-R	AACGTCCGTAGCTGTGAGTCCTG					59
RsPV11-CP-F	AAGGCTTGTCGGCTCTACA		contig315	CP	587	51
RsPV11-CP-R	TACCGTGCTGGTTCATCAC					51

RsRV11-Rd-F	TTCATCAAGAAGCATCAAGCC	Rhizoctonia solani RNA virus 11 (RsRV11)	contig14243	RdRp	690	50
RsRV11-Rd-R	CTCACCACCACGCATAATACC					54
RsRV11-HP-F	GCCAAGACCTCACAAACGG		contig11433	HP	972	53
RsRV11-HP-R	ACACCCAAACAATAGAACAAAAGA					51
