# Supplementary material for: Six Novel Mycoviruses Containing Positive Single-Stranded RNA and Double-Stranded RNA Genomes Co-Infect a Single Strain of the Rhizoctonia solani AG-3 PT
Source: Viruses. 2022 Apr 14;14(4):813. doi: 10.3390/v14040813 (PMC9025235; doi:10.3390/v14040813)
Supplement: Supplementary file 1 [file viruses-14-00813-s001.zip › Table S2.pdf]

Table S2. Primers used to validate the nucleotide sequences in the six mycoviruses (Rhizoctonia solani mitovirus 40, Rhizoctonia solani fusarivirus 4, Rhizoctonia solani fusarivirus 5, Rhizoctonia solani parititivirus 10, Rhizoctonia solani parititivirus 11, and Rhizoctonia solani RNA virus 11) based on the sequences obtained through metatranscriptome analysis of *Rhizoctonia solani* AG-3 PT strain ZJ-2H.

Mycovirus Name	Primer Name	Sequence (5'-3')	The Region of Polymerase Chain Reaction Product at Viral Genome (nt)	Annealing Temperature (°C)
Rhizoctonia solani mitovirus 40 (RsMV40)	RsMV40-1F	CTGATCTCCGCCCGTGTC	28-640	55
	RsMV40-1R	TCCCGTAAGCCAATCTACCTAT		53
	RsMV40-2F	GAATAGGTAGATTGGCTTACGGG	638-1372	55
	RsMV40-2R	GCTCTAAGCGAATCTTTGGTTTT		52
	RsMV40-3F	GTTGACAAACGGTCTGCCTAA	638-1799	52
	RsMV40-3R	GCTGCCCTACCTCGTAAAAGT		54
	RsMV40-4F	AAGTCAAGACAAGGCTATCAAGG	1587-2523	53
	RsMV40-4R	TGGCAACTTATACTCAAGCGAC		53
Rhizoctonia solani fusarivirus 4 (RsFV4)	RsFV4-1F	CGGGTCAGAGTCCGTTTTCG	881-1500	56
	RsFV4-1R	TCGGTCTTCCTCGGTTGTTTT		52
	RsFV4-2F	CATACACGGCAACATTCCACG	1679-2642	54
	RsFV4-2R	GACCAAGTCATCATCTTACTGTCATCA		57
	RsFV4-3F	AAGAGCGCCCTGTGCCTCAC	2416-3311	58
	RsFV4-3R	GCTTGACAAGTCGCCAACCCA		56
	RsFV4-4F	GGCTATTTCTGGCTGACGAGCTC	3935-4828	59
	RsFV4-4R	AATTCCTAAGAACCTTCGCGTATTTAA		54
	RsFV4-5F	GGAGGCTTACGAAAACAAACCG	3581-4502	55

	RsFV4-5R	TGTCAAGATGAGACGAACCCAGA		55
	RsFV4-6F	TTAGCGTTGGCTCTATCCCTCT	4141-5013	55
	RsFV4-6R	CCTTCGTAATCTTTGACCTTTTCG		54
	RsFV4-7F	GGTCAAAGATTACGAAGGCAAAC	5019-5966	53
	RsFV4-7R	CCCAAGTACAAAGCAACCATCACTAGAGAGTTAT		62
	RsFV4-8F	ACATCACATACCATGACCCGC	6302-7211	54
	RsFV4-8R	GAGCCACCAAATCACGAAAAC		52
	RsFV4-9F	GTTTTCGTGATTTGGTGGCTC	7211-8105	52
	RsFV4-9R	CTGGCTCTTCAATTTCTAGTTGCT		54
	RsFV4-10F	GAGTGACCGTGAGTGATGTGGA	7940-8876	57
	RsFV4-10R	GGCTTTTAGGCTTTTGCGTG		52
	RsFV4-11F	CGCAAAAGCCTAAAAGCCC	8878-9754	51
	RsFV4-11R	GTCACAACAGCCCAACAAACAC		55
	RsFV4-12F	TTCCAGTGCCAAGACCAAAAT	9629-10435	50
	RsFV4-12R	AACAGGCGCAACCTCTAAAGAC		55
Rhizoctonia solani fusarivirus 5 (RsFV5)	RsFV5-1F	ACAGACTCGGAGATTGAATATGTGAGG	182-1106	58
	RsFV5-1R	AGGTCATACCTGCTGCTGAAACG		57
	RsFV5-2F	CCGTTTCAGCAGCAGGTATG	1083-2732	54
	RsFV5-2R	GGCTCCAAATTCAGTTGACTCTT		53
	RsFV5-3F	ATGTCTACAACCAATCGCAAATC	1323-3196	52
	RsFV5-3R	GCAGTGGATGCTCGTGAACC		56
	RsFV5-4F	GCGAGAATTGTGACATGGGTTCA	3179-3826	55
	RsFV5-4R	TGACAAGGCCAGGCAACAGAG		56
	RsFV5-5F	ACTGAAGAACGGGCGACG	3261-4190	53
	RsFV5-5R	CCATAACACGATAGGGCTGACT		55
	RsFV5-6F	GCTTATCCTTGCATCTGTGGTGTTAGTCAGCCCTAT	4166-5138	66

	RsFV5-6R	CCTGTCAAATTCATGCTGGACGT		55
	RsFV5-7F	TAGCCAAAGTCATAAAACAAGTCA	4393-6065	51
	RsFV5-7R	ATGTGGTCATCGCCATACACT		52
	RsFV5-8F	ACGCTTACACCATACCCACTTT	5488-6918	53
	RsFV5-8R	TGCCTGTTCTCAACGCCAT		51
	RsFV5-9F	CCCGACTGAGGAAGAAGGCACA	6602-7369	59
	RsFV5-9R	GCAAGTGATACTCTAGGCACCACAAC		60
	RsFV5-10F	ACAACATCATCAGCGGGTCA	7309-8082	52
	RsFV5-10R	CTTTTCCTCTAAACTGGACAATACA		53
Rhizoctonia solani partitivirus (RsPV10)	RsPV10-Rd-1F	CACGAAACGCCCTTCAACA	162-1134	51
	RsPV10-Rd-1R	GGGGTAGGAGACAGTCGGAAC		58
	RsPV10-Rd-2F	ATTCATTTGTTACCCTTGACTGG	1024-1787	52
	RsPV10-Rd-2R	GTCTGGTGAGTTTCCGAATACC		55
	RsPV10-CP-1F	TCGTCGTGGAATAGGTGGAA	219-1039	52
	RsPV10-CP-1R	CGATGGGCGTGTTGAAGTA		51
	RsPV10-CP-2F	AAGCCATCTTTACCGACCG	953-1623	51
	RsPV10-CP-2R	ATTGGGAAGGATTGACACGA		50
	RsPV10-CP-3F	TTGTCACCGTCACCACCG	1130-1786	53
	RsPV10-CP-3R	AAGCGAGAAAGATCATTGTTAGAG		52
Rhizoctonia solani partitivirus 11 (RsPV11)	RsPV11-Rd-1F	TAACTCCTCGTTCTGAACCTCCA	25-567	55
	RsPV11-Rd-1R	CGAATTTGTTGCCGTGCC		50
	RsPV11-Rd-2F	TGGGCACGGCAACAAA	548-1321	46
	RsPV11-Rd-2R	CGATGAGTAAACCAAGACGGATG		55
	RsPV11-Rd-3F	CTATCATCCGTCTTGGTTTACTCATC	1318-1835	55
	RsPV11-Rd-3R	GTAGTCGTCAGGCCAGGTGC		58
	RsPV11-CP-1F	TCCACCGTCCCCGCAAAG	130-887	55

	RsPV11-CP-1R	CGTGGTGAGGGATGTCGTTCT		56
	RsPV11-CP-2F	CCAGCCGACTGACCACAA	843-1750	53
	RsPV11-CP-2R	ATTCATAACAGAAGAGGCAAACAT		51
Rhizoctonia solani RNA virus 11 (RsRV11)	RsRV11-Rd-1F	CTAAGGTGCCAGTCTGGTCTATC	15-460	57
	RsRV11-Rd-1R	CGGTTGTCTTGACCTTGTTCTT		53
	RsRV11-Rd-2F	GGAAGACGATCCAGGAAGAAC	445-1408	54
	RsRV11-Rd-2R	TGCCATCATCCCGAAAGAC		51
	RsRV11-Rd-3F	CACTTATGCCGTGGTTGATTC	1286-2056	52
	RsRV11-Rd-3R	CAAACTGTGCCTTCCTGAGAC		54
	RsRV11-HP-1F	AACTAAGGTGCCAGTCGGGTCTATTCAA	11-907	60
	RsRV11-HP-1R	GGCGGTTCCTTCTTTCTTCTGGTGTAT		60
	RsRV11-HP-2F	CCAGAAGAAAGAAGGAACCGCCAATA	886-1608	58
	RsRV11-HP-2R	GGAGGAAAGAATACGGTCTTACTTTGTAGCACA		62
