# Supplementary material for: Six Novel Mycoviruses Containing Positive Single-Stranded RNA and Double-Stranded RNA Genomes Co-Infect a Single Strain of the Rhizoctonia solani AG-3 PT
Source: Viruses. 2022 Apr 14;14(4):813. doi: 10.3390/v14040813 (PMC9025235; doi:10.3390/v14040813)
Supplement: Supplementary file 1 [file viruses-14-00813-s001.zip › Table S3.pdf]

Table S3. Primers used to determine the terminal sequences of the six mycoviruses (Rhizoctonia solani mitovirus 40, Rhizoctonia solani fusarivirus 4, Rhizoctonia solani fusarivirus 5, Rhizoctonia solani parititivirus 10, Rhizoctonia solani parititivirus 11, and Rhizoctonia solani RNA virus 11) associated with *Rhizoctonia solani* AG-3 PT strain ZJ-2H by rapid amplification of cDNA ends (RACE).

Primer Name	Sequence (5'-3')	Amplification Target	Annealing Temperature (°C)
RsMV40-5'	GATTACGCCAAGCTTGGGGTCGCATTGCACTTTGTGTA	5'-UTR of Rhizoctonia solani mitovirus 40 (RsMV40)	68
RsMV40-3'	GATTACGCCAAGCTTTTCTCGTCGCTTGAGTATAAGTTGCC	3'-UTR of RsMV40	68
RsFV4-5'	GATTACGCCAAGCTTTGTTGTCACTCTCATCCCCCAGTCTTC	5'-UTR of Rhizoctonia solani fusarivirus 4 (RsFV4)	69
RsFV4-3'	GATTACGCCAAGCTTGGGCTGTTGTGACTGAAGAATCGG	3'-UTR of RsFV4	69
RsFV5-5'	GATTACGCCAAGCTTGCTTGAATAGAGACACATCGTCGTGAATC	5'-UTR of Rhizoctonia solani fusarivirus 5 (RsFV5)	68
RsFV5-3'	GATTACGCCAAGCTTGGAGTGGAACATTGAGATGCGAGA	3'-UTR of RsFV5	68
RsPV10-Rd-5'	GATTACGCCAAGCTTTTGTGCGGGTGGGTTGTTGAAGGGC	5'-UTR of double-stranded RNA (dsRNA) 1 segment of Rhizoctonia solani parititvirus 10 (RsPV10)	71
RsPV10-Rd-3'	GATTACGCCAAGCTTGATGTGAAGTCCTCAGTTATCGTAATGCG	3'-UTR of dsRNA1 of RsPV10	68
RsPV10-CP-5'	GATTACGCCAAGCTTCCAGTGATTCCACCTATTCCACGACGA	5'-UTR of dsRNA2 of RsPV10	69
RsPV10-CP-3'	GATTACGCCAAGCTTCCCTCAATCTTGTCACCGTCACCACC	3'-UTR of dsRNA2 of RsPV10	71
RsPV11-Rd-5'	GATTACGCCAAGCTTGCCAGCGACGAGTCCACTGAAATA	5'-UTR of dsRNA1 of Rhizoctonia solani parititvirus 11 (RsPV11)	69
RsPV11-Rd-3'	GATTACGCCAAGCTTGTTAGCACAACTCTACCACACGAAGGC	3'-UTR of dsRNA1 of RsPV11	69

RsPV11-CP-5'	GATTACGCCAAGCTTTGAGGGATGTCGTTCTCGGCAATGA	5'-UTR of dsRNA2 of RsPV11	69
RsPV11-CP-3'	GATTACGCCAAGCTTGTGGATTCGCCTTCGCTGACGCTA	3'-UTR of dsRNA2 of RsPV11	70
RsRV11-Rd-5'	GATTACGCCAAGCTTCATTAGCAAACAAACACCTCACA	5'-UTR of dsRNA1 of Rhizoctonia solani RNA Virus (RsRV11)	64
RsRV11-Rd-3'	GATTACGCCAAGCTTGATGGAATCGTGGATTGTTATGT	3'-UTR of dsRNA1 of RsRV11	64
RsRV11-HP-5'	GATTACGCCAAGCTTGGCGGTTCCTTCTTTCTTCTGGTGTATCT	5'-UTR of dsRNA2 of RsRV11	69
RsRV11-HP-3'	GATTACGCCAAGCTTCACGGACTTGTCTTGAGGTGAGTTCTGC	3'-UTR of dsRNA2 of RsRV11	70
