# Supplementary material for: Six Novel Mycoviruses Containing Positive Single-Stranded RNA and Double-Stranded RNA Genomes Co-Infect a Single Strain of the Rhizoctonia solani AG-3 PT
Source: Viruses. 2022 Apr 14;14(4):813. doi: 10.3390/v14040813 (PMC9025235; doi:10.3390/v14040813)
Supplement: Supplementary file 1 [file viruses-14-00813-s001.zip › Table S5.pdf]

Table S5. The reference viruses of families *Hypoviridae*, *Mitoviridae*, *Narnaviridae*, *Partitiviridae*, *Megabinaviridae*, and *Totiviridae*, and proposed families Fusariviridae and Bipartitiviridae, which were retrieved from GenBank and used to conduct phylogenetic analysis.

Family	Name of the Reference Viruses	Abbreviation	Host	GenBank Accession Numbers
Fusariviridae	Neurospora crassa fusarivirus 1-JW222	NcFV1-JW222	*Neurospora crassa*	BCL64190.1
	Neurospora intermedia fusarivirus 1	NiFV1	*Neurospora intermedia*	BCB17077.1
	Neurospora crassa fusarivirus 1-JW45	NcFV1-JW45	*Neurospora crassa*	LC586023
	Neurospora crassa fusarivirus 1- JW164	NcFV1-JW164	*Neurospora crassa*	LC586023
	Neurospora discreta fusarivirus 2-W683	NdFV2-W683	*Neurospora discreta*	BCL64194.1
	Nigrospora oryzae fusarivirus 1	NoFV1	*Nigrospora oryzae*	KU980909
	Sclerotinia sclerotiorum fusarivirus 1	SsFV1	*Sclerotinia sclerotiorum*	YP_009143301.1
	Alternaria brassicicola fusarivirus 1	AbFV1	*Alternaria brassicicola*	YP_009222009.1
	Alternaria alternata fusarivirus 1	AaFV1	*Alternaria alternata*	BBD71147.1
	Botrytis cinerea fusarivirus 1	BcFV1	*Botrytis cinerea*	YP_009480678.1
	Rhizoctonia solani fusarivirus virus 2	RsFV2	*Rhizoctonia solani*	MK558256.1
	Rhizoctonia solani fusarivirus virus 1	RsFV1	*Rhizoctonia solani*	QDW92695.1
	Rhizoctonia solani fusarivirus virus 3	RsFV3	*Rhizoctonia solani*	MK558258.1
	Macrophomina phaseolina single-stranded RNA virus 1	MpRV1	*Macrophomina phaseolina*	KP900890
	Penicillium aurantiogriseum fusarivirus 1	PaFV1	*Penicillium aurantiogriseum*	NC_028467
	Penicillium roqueforti ssRNA mycovirus 1	PrMV1	*Penicillium roqueforti*	NC_024699
	Fusarium graminearum dsRNA mycovirus 1	FgV1	*Fusarium graminearum*	NC_006937
	Pleospora typhicola fusarivirus 1	PtFV1	*Pleospora typhicola*	NC_028470
	Rosellinia necatrix fusarivirus 1	RnFV1	*Rosellinia necatrix*	YP_009047147.1
	Fusarium poae fusarivirus 1	FpFV1	*Fusarium poae*	YP_009272906.1
	Neurospora discreta fusarivirus 1-W683	NdFV1-W683	*Neurospora discreta*	BCL64192.1
Hypoviridae	Cryphonectria hypovirus 1	CHV1	*Cryphonectria parasitica*	NP_041091.1

	Cryphonectria hypovirus 2	CHV2	*Cryphonectria parasitica*	AAA20137.1
	Rhizoctonia solani mitovirus 11	RsMV11	Rhizoctonia solani	ALD89116.1
	Rhizoctonia solani mitovirus 34	RsMV34	Rhizoctonia solani	QDW65424.1
	Soybean leaf-associated mitovirus 1	SlaMV1	Soybean phyllosphere fungi	ALM62241.1
	Rhizoctonia solani mitovirus 2	RsMV2	Rhizoctonia solani	ALD89121.1
	Botryosphaeria dothidea mitovirus 1	BdMV1	Botryosphaeria dothidea	QMU24933.1
	Buergenerula spartinae mitovirus 1	BsMV1	Buergenerula spartinae	AHY03257.1
	Setosphaeria turcica mitovirus 1	StMV1	Setosphaeria turcica	AZT88625.1
	Plasmopara viticola lesion associated mitovirus 43	PvaMV43	Plasmopara viticola	QIR30266.1
	Plasmopara viticola lesion associated mitovirus 46	PvaMV46	Plasmopara viticola	QIR30269.1
	Sclerotinia sclerotiorum mitovirus 7-A	SsMV7-A	Sclerotinia sclerotiorum	AWY10969.1
	Fusarium poae mitovirus 4	FpMV4	Fusarium poae	YP_009272901.1
	Botrytis cinerea mitovirus 1	BcMV1	Botrytis cinerea	CEZ26300.1
Mitoviridae	Bremia lactucae associated mitovirus 1	BlaMV1	Bremia lactucae	QIP68024.1
	Sclerotinia sclerotiorum mitovirus 3-NZ1	SsMV3/NZ1	Sclerotinia sclerotiorum	AGC24232
	Sclerotinia sclerotiorum mitovirus 12	SsMV12	Sclerotinia sclerotiorum	AHF48628.1
	Macrophomina phaseolina mitovirus 1	MpMV1	Macrophomina phaseolina	ALD89100.1
	Sclerotinia sclerotiorum mitovirus 6-14563	SsMV6/14563	Sclerotinia sclerotiorum	AHX84133
	Rhizoctonia solani mitovirus 35	RsMV35	Rhizoctonia solani	QDW80891.1
	Fusarium boothii mitovirus 1	FbMV1	Fusarium boothii	BBG56022.1
	Fusarium poae mitovirus 3	FpMV3	Fusarium poae	YP_009272900.1
	Alternaria alternata mitovirus 1	AaMV1	Alternaria alternata	QDB74990.1
	Rhizoctonia solani mitovirus 38	RsMV38	Rhizoctonia solani	QDW65426.1
	Epicoccum nigrum mitovirus 1	EnMV1	Epicoccum nigrum	QDB74989.1
	Rhizoctonia solani mitovirus 22	RsMV22	Rhizoctonia solani	QDW80890.1
	Ophiostoma mitovirus 7	OnuMV7	Ophiostoma novo-ulmi	KF031943.1

	Rhizoctonia mitovirus 1	RsMV1	Rhizoctonia solani	AHL25281
	Tuber excavatum mitovirus	TeMV	Tuber excavatum	AEP83726
	Sclerotinia sclerotiorum mitovirus 2-KL-1	SsMV2/KL-1	Sclerotinia sclerotiorum	AEX91879
	Sclerotinia sclerotiorum mitovirus 4-NZ1	SsMV4/NZ1	Sclerotinia sclerotiorum	AGC24233
	Sclerotinia sclerotiorum mitovirus 1-KL-1	SsMV1/KL-1	Sclerotinia sclerotiorum	AEX91878
	Sclerotinia sclerotiorum mitovirus 5-11691	SsMV5/11691	Sclerotinia sclerotiorum	AHX84130
Narnaviridae	Saccharomyces 23S RNA narnavirus	ScNV23s	Saccharomyces cerevisiae	NP_660177
	Saccharomyces 20S RNA narnavirus	ScNV20s	Saccharomyces cerevisiae	NP_660178
Proposed Bipartitiviridae	Cryphonectria parasitica bipartite mycovirus 1	CpBV1	Cryphonectria parasitica	YP_007985675.1
	Botryosphaeria dothidea bipartite mycovirus 1	BdBMV1	Botryosphaeria dothidea	QUP79399.1
	Curvularia virus 2	CuV2	Curvularia	ALO61392.1
	Corynespora cassiicola bipartite mycovirus 1	CcBMV1	Corynespora cassiicola	QNC69629.1
	Trichoderma harzianum bipartite mycovirus 1	ThBV1	Trichoderma harzianum	YP_009553330.1
	Trichoderma harzianum bipartite mycovirus 1	ThBMV1	Trichoderma harzianum	AXU24203.1
	Curvularia thermal tolerance virus	CtTV	Curvularia	YP_001976143.1
	Myriodontium keratinophilum bipartite virus 1	MkBV1	Myriodontium keratinophilum	AYP71809.1
	Penicillium aurantiogriseum bipartite virus 1	PaBV1	Penicillium aurantiogriseum	YP_009182335.1
	Sclerotium hydrophilum virus 1	ShV1	Sclerotium hydrophilum	YP_009273017.1
	Lactarius tabidus RNA virus 1	LtRV1	Lactarius tabidus	AMK47915.3
	Gremmeniella abietina RNA virus 6	GaRV6	Gremmeniella abietina	AIU98624.1
	Fusarium graminearum dsRNA mycovirus 5	FgRV5	Fusarium graminearum	APP91269.1
	Fusarium graminearum dsRNA mycovirus 4	FgRV4	Fusarium graminearum	YP_003288790.1
	Heterobasidion RNA virus 6	HeRV6	Heterobasidion annosum	ADW82833.1
	Sclerotium rolfsii unassigned dsRNA virus 2	SrRV2	Sclerotium rolfsii	AZF86116.1
	Sclerotium rolfsii unassigned dsRNA virus 1	SrRV1	Sclerotium rolfsii	AZF86115.1
	Rhizoctonia fumigata mycovirus	RfMV	Rhizoctonia fumigata	YP_009134757.1

	Rhizoctonia solani dsRNA virus 1	RsRV1	Rhizoctonia solani	AFZ85210.1
	Rhizoctonia solani mycovirus 2	RsMV2	Rhizoctonia solani	ANR02696.1
	Rhizoctonia solani bipartite-like virus 1	RsBLV1	Rhizoctonia solani	QDW81299.1
	Ceratobasidium mycovirus-like	CeMVL	Ceratobasidium sp.	AOX47586.1
Partitiviridae	Ustilaginoidea virens partitivirus 3	UvPV3	Ustilaginoidea virens	YP_008327312.1
	Ustilaginoidea virens partitivirus 2	UvPV2	Ustilaginoidea virens	YP_008327313.1
	Fusarium solani virus 1	FsV1	Fusarium solani	BAA09520.1
	Gremmeniella abietina RNA virus MS1	GaRVMS1	Gremmeniella abietina	AAM12240.1
	Penicillium stoloniferum virus S	PsVS	Penicillium stoloniferum	CAJ01909.1
	Aspergillus fumigatus partitivirus 1	AfPV1	Aspergillus fumigatus	CAY25801.2
	Fusarium poae virus 1	FpV1	Fusarium poae	AAC98734.1
	Rhizoctonia solani virus 717	RsV717	Rhizoctonia solani	AAF22160.1
	Sclerotinia sclerotiorum partitivirus 1	SsPV1	Sclerotinia sclerotiorum	AFR78160.1
	Rosellinia necatrix partitivirus 3	RnPV3	Rosellinia necatrix	BAM36401.1
	Rosellinia necatrix partitivirus 4	RnPV4	Rosellinia necatrix	BAM36402.1
	Heterobasidion partitivirus 2	HePV2	Heterobasidion parviporum	ADL66905.1
	Rosellinia necatrix partitivirus 2	RnPV2	Rosellinia necatrix	BAM78602.1
	Vicia faba partitivirus 1	VfPV1	Vicia faba	ABJ99996.1
	Sclerotinia sclerotiorum partitivirus S	SsPVS	Sclerotinia sclerotiorum	YP 003082248.1
	Heterobasidion partitivirus 3	HePV3	Heterobasidion ecrustosum	ACO37245.1
	Heterobasidion partitivirus 1	HePV1	Heterobasidion abietinum	ADV15441.1
	Rhizoctonia fumigata partitivirus	RfPV	Rhizoctonia fumigata	AJE25830.1
	Gaeumannomyces tritici partitivirus 2	GtPV2	Gaeumannomyces tritici	AZT88604.1
	Raphanus sativus cryptic virus 4	RsCV4	Raphanus sativus	ATG29853.1
	Vicia cryptic virus	VCV	Vicia faba	ABN71239.1
	Beet cryptic virus 1	BCV1	Beta vulgaris	ACA81389.1

	White clover cryptic virus 1	WCCV1	white clover	AAU14888.1
	Red clover cryptic virus 1	RccV1	Red clover	YP_008719882.1
	Fig cryptic virus	FcV	Fig	CBW77436.1
	Beet cryptic virus 3	BcV3	Beet	AAB27624.1
	Pepper cryptic virus 2	PcV2	Pepper	AEJ07892.1
	Beet cryptic virus 2	BcV2	Beet	ADP24757.1
	Pepper cryptic virus 1	PcV1	Pepper	AEJ07890.1
Megabinaviridae	Botrytis porri botybirnavirus 1	BpBV1	Botrytis porri	AFD04798.1
	Rosellinia necatrix megabirnavirus 1	RnMBV1	Rosellinia necatrix	BAI48016.1
	Pleosporales megabirnavirus 1	PmBV1	Pleosporales sp.	ALO50147.1
	Sclerotinia sclerotiorum megabirnavirus 1	SsMBV1	Sclerotinia sclerotiorum	YP_009143529.1
Totiviridae	Botryotinia fuckeliana totivirus 1	BfTV1	Botryotinia fuckeliana	YP_001109580.1
	Gremmeniella abietina RNA virus L1	GaRvL1	Gremmeniella abietina	NP_624332.2
	Gremmeniella abietina RNA virus L2	GaRvL2	Gremmeniella abietina	YP_044807.1
	Aspergillus mycovirus 178	AsMV178	Aspergillus nidulans	ABX79995.1
	Penicillium aurantiogriseum totivirus 1	PaTV1	Penicillium aurantiogriseum	YP_009212848.1
	Magnaporthe oryzae virus 2	MoV2	Magnaporthe oryzae	YP_001649206.1
	Sphaeropsis sapinea RNA virus 2	SsRV2	Sphaeropsis sapinea	NP_047560.1
