# Supplementary figures and images for: Six Novel Mycoviruses Containing Positive Single-Stranded RNA and Double-Stranded RNA Genomes Co-Infect a Single Strain of the Rhizoctonia solani AG-3 PT
Source: Viruses. 2022 Apr 14;14(4):813. doi: 10.3390/v14040813 (PMC9025235; doi:10.3390/v14040813)

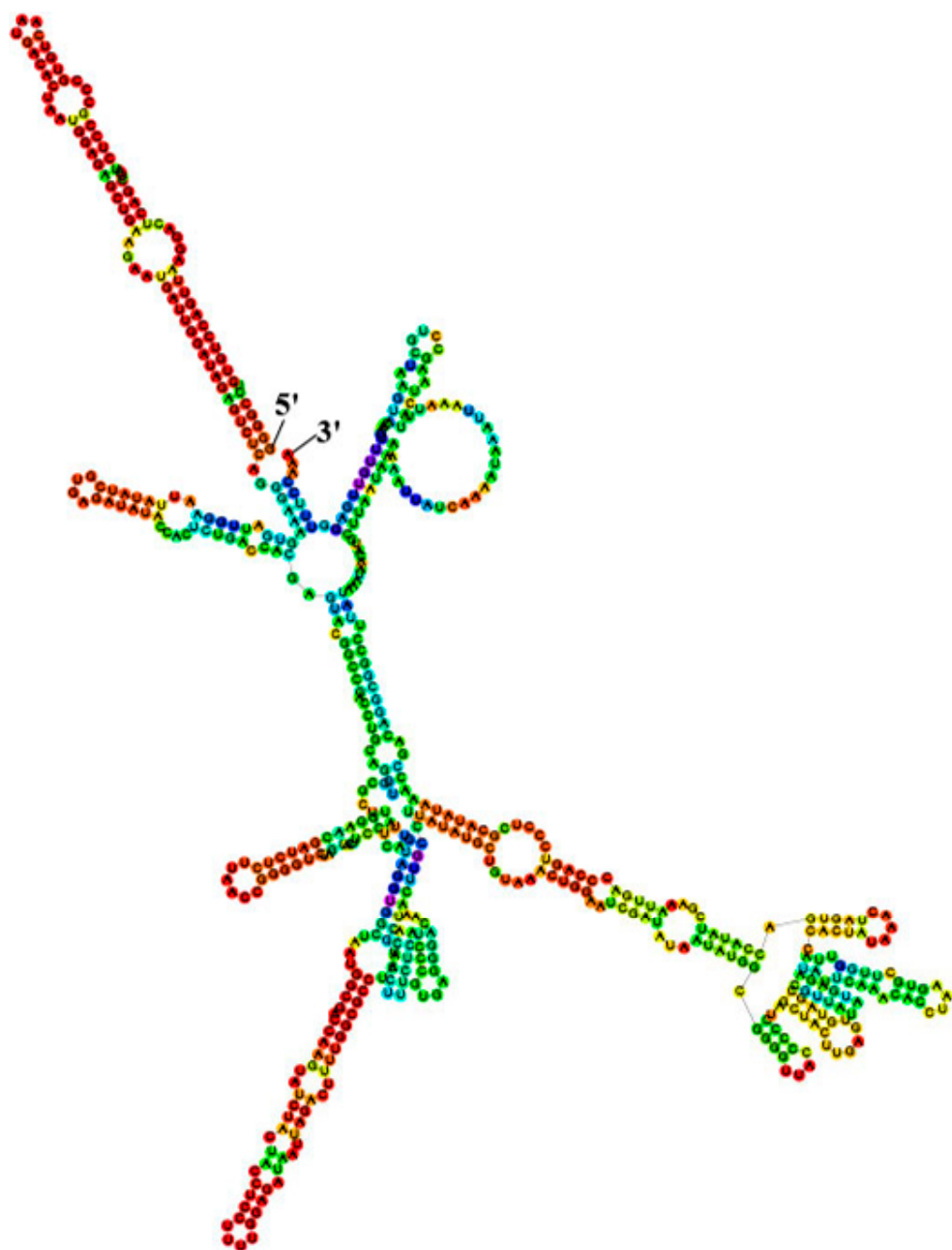

**RsMV40, 5'-terminus**  
 **$\Delta G = -130.60$  kcal/mol**

Supplement: Supplementary file 1 [file viruses-14-00813-s001.zip › Figure S2.pdf]

**A**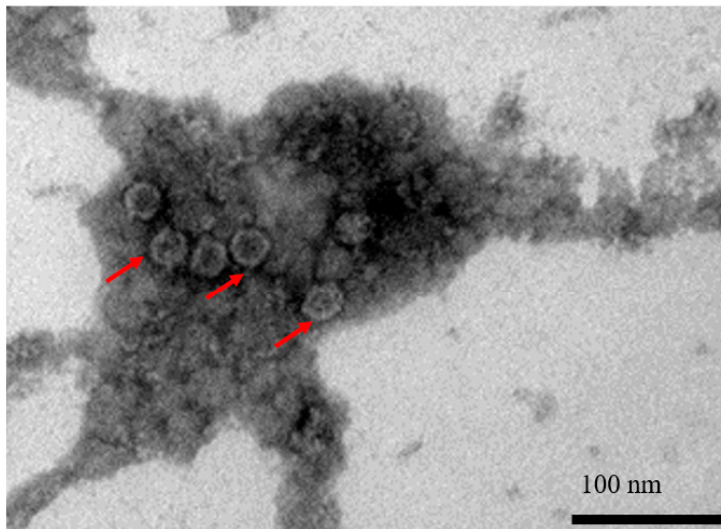**B**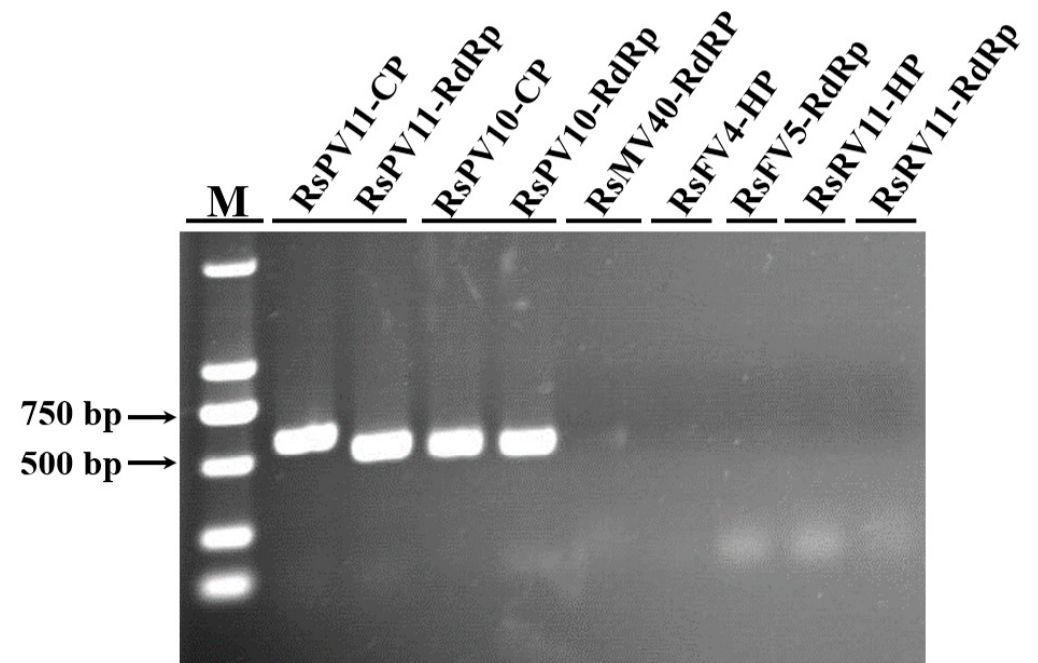

Supplement: Supplementary file 1 [file viruses-14-00813-s001.zip › Figure S3.pdf]
